# Neurofibromatosis type 1: an illustrative case of cutaneous manifestations

**DOI:** 10.11604/pamj.2025.52.140.49312

**Published:** 2025-12-04

**Authors:** Pawan Banduji Itankar, Gaurav Rajendra Sawarkar

**Affiliations:** 1 Department of Rachana Sharir, Mahatma Gandhi Ayurved College, Hospital and Research Centre, Datta Meghe Institute of Higher Education and Research (Deemed to be University), Salod (H), Wardha, Maharashtra, India

**Keywords:** Neurofibromin, neurofibromatosis, nerve sheath tumors

## Image in medicine

A 36-year-old male, working as a shopkeeper, presented with multiple soft, skin-coloured swellings on the chest and upper back that had progressively increased in number over the past 7 years. The lesions were asymptomatic, though he expressed cosmetic concern and mild discomfort when lying on his back. He reported that the swellings were initially few and small but gradually became more prominent over time. The patient denied any pain, discharge, ulceration, or associated itching. There was no history of trauma, recent infection, or new medication use. He had not sought medical care previously, assuming the lesions were harmless. There was no known family history of cancer or neurological conditions. On clinical examination, multiple soft, dome-shaped nodules ranging from 0.5 to 4 cm in size were seen scattered across the anterior chest and upper back. There were no signs of secondary infection, ulceration, or malignancy. Based on the characteristic skin findings, ocular signs, family history, and clinical criteria, a diagnosis of neurofibromatosis type 1 (NF1) was made. Neurofibromatosis type 1 is a genetic neurocutaneous disorder caused by mutations in the NF1 gene on chromosome 17, which encodes the tumor suppressor protein neurofibromin. It follows an autosomal dominant inheritance pattern with complete penetrance and variable expressivity. The presence of multiple cutaneous neurofibromas, *café-au-lait macules*, axillary/ inguinal freckling, and Lisch nodules characterises the condition. It may also involve the central nervous system, bones, and vasculature, with potential complications including optic gliomas and malignant peripheral nerve sheath tumors.

**Figure 1:**
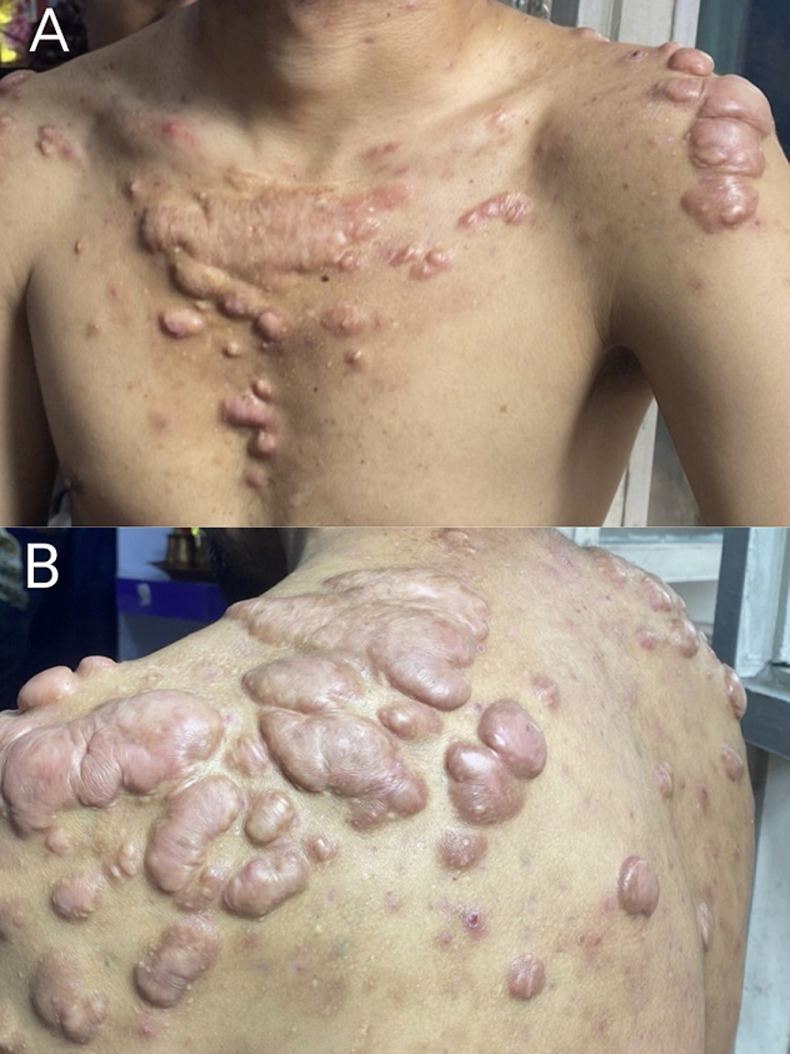
A,B) multiple soft, skin-colored neurofibromas on the chest, and dome-shaped neurofibroma nodules over the upper back region

